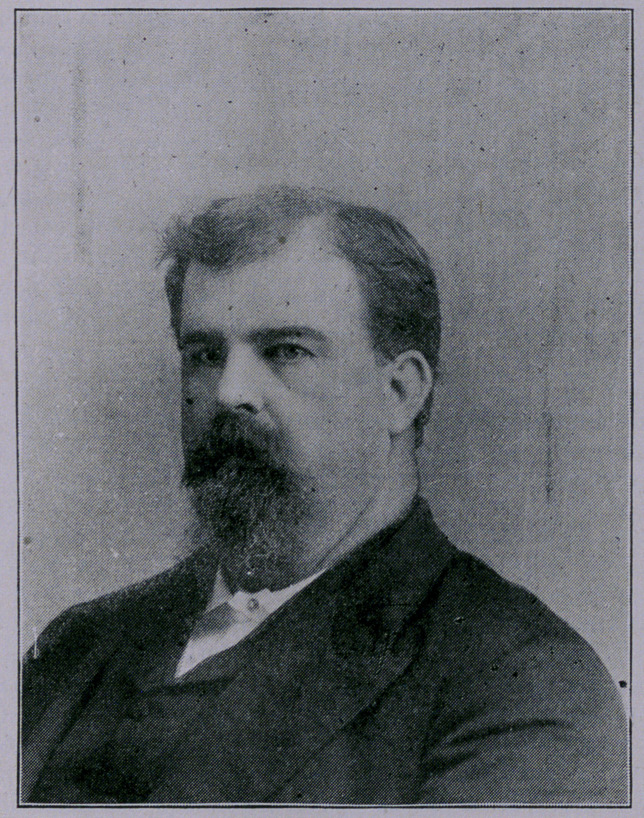# Death of Dr. F. R. Martin

**Published:** 1907-10

**Authors:** 


### DEATH OF Dr. F. R. MARTIN.

Our esteemed colleague and beloved friend of many years, Dr. F. R. Martin, of Kyle, died in Austin, Wednesday, September 18 (ult.), aged 62. The remains were interred at his home, Kyle, Texas. Dr. Martin was born in Copiah county, Miss., and practiced medicine there prior to his removal to Texas in 1875. All who knew him loved him, and "none named him but to praise." Pure in heart, gentle as a woman, as courageous as a lion, he was a splendid type of the ethical physician, and his death will be mourned by a wide circle of attached friends. Nearly two years ago he lost his oldest son, Dr. Chaille Martin, of the A. & M. College, and it literally broke his heart. Shortly afterwards he suffered an apoplexy, from the effects of which he never fully recovered. I append a brief biographical sketch. I knew him well and loved him.

He was a man of unusual ability and imposing appearance. He was born near Hazelhurst, Miss., reared on a plantation, educated at the best private and public schools, and graduated in medicine from Tulane University, New Orleans, in 1873. He located at Elgin in 1875, where he resided nine years, during which he married, January 14, 1877, Miss Mary A. Davis, of Bastrop county. He removed later to Kyle, where he resided twenty-three years, honored and respected by all who knew him. He was distinctly a leading physician, having served as physician of the Houston and Texas Central railroad and practiced widely. He was one of the oldest members of the Texas Medical Association and a charter member of the Austin District Medical Association, of which he was some time president. He was also a contributor to the medical publications to which he furnished some most acceptable papers.

Dr. Martin is survived by his wife, two sons and five daughters, who are highly esteemed in the community.

---

THERE OCCURRED two or more rather exasperating typographical errors in our September number, for which I want to apologize; one was "*montes parturient*" for "*montes parturiunt*," the other "delapidated" for "dilapidated." Give me credit for knowing how to spell, at least, and blame the erorrs on the blamed hot weather.

## Figures and Tables

**Figure f1:**